# Alternative polyadenylation and splicing regulation in refractory juvenile dermatomyositis: CELF2 at the crossroads

**DOI:** 10.1016/j.omtn.2025.102725

**Published:** 2025-10-13

**Authors:** Riccardo Varrica, Laura Lentini

**Affiliations:** 1 Department of Biological, Chemical and Pharmaceutical Sciences and Technologies (STEBICEF), University of Palermo, Palermo, Italy

Juvenile dermatomyositis (JDM) is a rare, chronic autoimmune disease characterized by muscle weakness, skin rashes, and systemic inflammation. While corticosteroids and immunosuppressants remain the cornerstone of therapy, a significant subset of patients develop refractory disease, experiencing recurrent relapses and accumulating disability. For these patients, autologous hematopoietic stem cell transplantation (AHSCT) has emerged as a last-resort option, yet its molecular mechanisms of action remain poorly defined.

In their recent study, Wei, Zhang, Lu, and colleagues provide the first transcriptome-wide analysis of alternative polyadenylation (APA) and alternative splicing (AS) in refractory JDM before and after AHSCT.^1^

The authors identify a striking switch in polyadenylation site (PAS) usage within the RNA-binding protein CUGBP Elav-like family member 2 (CELF2). In refractory JDM, CELF2 predominantly utilizes a proximal PAS, whereas recovery after AHSCT is associated with a shift to distal PAS usage. This transition not only enhances CELF2 expression but also reshapes the splicing landscape of immune-related genes. Among them, Cathepsin B (CTSB) undergoes a novel exon insertion event induced by proximal PAS usage of CELF2, linking APA and splicing dysregulation to tumor necrosis factor (TNF) signaling and treatment resistance.

These findings uncover a previously unrecognized regulatory axis in JDM and suggest new molecular entry points for therapeutic intervention.

RNA processing is increasingly recognized as a dynamic regulator of immune responses. Both AS and APA expand transcript diversity, fine-tune protein isoform expression, and determine RNA stability by altering microRNA-binding sites.^2^^,^^3^ In T cells, splicing governs antigen receptor signaling, cytokine responsiveness, and differentiation.^4^ Likewise, APA contributes to immune activation by shortening 3′ UTRs and releasing transcripts from microRNA repression.^5^ Despite this, the contribution of these mechanisms to autoimmune pathology remains underexplored.

In dermatomyositis, previous studies had associated specific splicing events with disease subtypes and clinical manifestations.^6^ However, global APA dynamics had never been investigated. By combining polyadenylation sequencing and RNA sequencing analysis, Wei et al.^1^ offer a unique window into the post-transcriptional remodeling that accompanies clinical remission after AHSCT. Their work supports the notion that immune dysfunction in JDM is not only the result of genetic predisposition and inflammatory signaling but also of misregulated RNA processing. This observation opens new, fundamental scenarios in the comprehension of the immune pathologies and a possible way for new therapies.

The central finding of the study is the preferential usage of proximal PAS in CELF2 in refractory JDM.^1^ CELF2 is a splicing regulator with established roles in T cell activation.^7^ By selecting the proximal PAS, CELF2 transcripts acquire shorter 3′ UTRs, leading to reduced expression due to the loss of stabilizing elements and increased microRNA-mediated repression. In contrast, distal PAS usage restores higher CELF2 expression.

Functionally, this APA choice has profound consequences. Proximal PAS usage correlates with enhanced TNF signaling, elevated TNF-α levels, and resistance to methotrexate/cyclophosphamide therapy. Indeed, patients with refractory JDM harboring proximal PAS usage of CELF2 showed persistent inflammation and calcinosis. At the same time, AHSCT shifted PAS selection to the distal site, lowering TNF activity and enabling clinical recovery.^1^^,^^8^ This mechanistic link provides a molecular explanation for why conventional immunosuppression fails in certain patients while stem cell transplantation succeeds.

Beyond CELF2 expression, APA-driven changes in CELF2 profoundly reshape the splicing landscape. The authors identify over 6,000 AS events in refractory JDM, with immune-related genes disproportionately affected.^1^ Among these, CTSB emerges as a key target. Proximal PAS usage of CELF2 induces a novel insertion of exons 2–3 in CTSB, generating isoforms absent in healthy or responsive JDM cases. CTSB, a cysteine protease implicated in antigen processing and inflammasome activation, interacts with TNF-α signaling.^9^ The aberrant isoform appears “toxic” in the context of refractory disease, perpetuating immune activation and tissue damage.

It is also worth noting that APA changes were not limited to CELF2: additional splicing regulators such as SRSF10 exhibited altered PAS usage, indicating that CELF2 is part of a broader network of RNA-binding proteins affected in JDM. Moreover, loss of distal 3′-UTR segments frequently removes conserved microRNA-binding sites, further contributing to transcript destabilization and deregulated protein expression (Figure 1). This suggests a multilayered mechanism in which APA influences both splicing outcomes and post-transcriptional repression. The study by Wei et al.^1^ has several implications for the field of RNA therapeutics and autoimmunity.

(1) Biomarker potential: proximal PAS usage of CELF2 could serve as a molecular biomarker to stratify JDM patients at risk of refractoriness and poor response to standard immunosuppression.
(2) Therapeutic targeting: interventions aimed at modulating PAS usage—whether by antisense oligonucleotides, CRISPR-mediated editing, or small molecules targeting cleavage/polyadenylation complexes—may restore distal PAS usage and normalize CELF2 function.^3^^,^^10^
(3) RNA-guided therapies in autoimmunity: while RNA therapeutics are well established in genetic diseases, this study extends their relevance to immune-mediated disorders, supporting a broader translational vision.
(4) Intersection with TNF blockade: the link between CELF2 APA, CTSB splicing, and TNF signaling explains why anti-TNF therapy (e.g., adalimumab) shows limited efficacy in some refractory JDM cases.^1^^,^^8^ Understanding RNA-level regulation may refine therapeutic combinations.
(5) APA profiling as a diagnostic tool: incorporating polyadenylation profiling into clinical practice could identify refractory patients earlier and guide personalized interventions.

**Figure 1:**
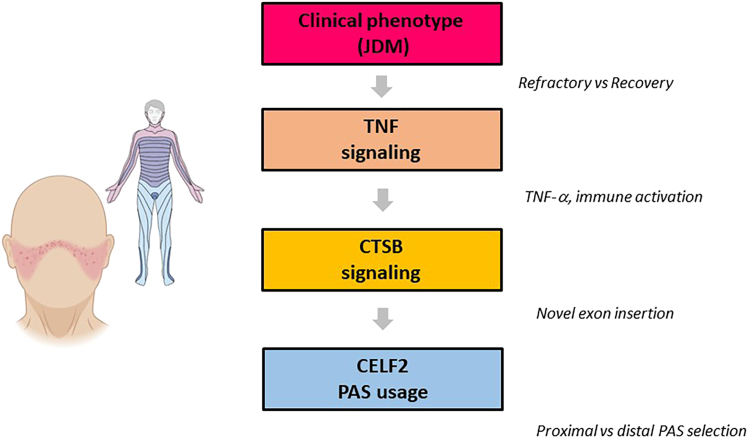
Mechanistic model of refractory juvenile dermatomyositis Proximal PAS usage of CELF2 drives aberrant CTSB splicing and sustained TNF signaling; distal PAS usage after AHSCT restores transcript balance and promotes clinical recovery.

While provocative, the study also raises important questions. The analysis relies on a limited number of refractory JDM patients, with detailed mechanistic insights derived from one individual undergoing AHSCT. Larger cohorts will be essential to validate CELF2 PAS usage as a generalizable feature. Moreover, while luciferase and *in vitro* assays confirm the functional impact of APA shifts, the precise molecular machinery governing PAS choice in CELF2 remains undefined.

Another open issue concerns the specificity of these findings. Is CELF2 PAS switching unique to JDM, or does it occur across autoimmune diseases where TNF signaling is central, such as lupus or rheumatoid arthritis^9^? Similarly, is CTSB splicing the dominant effector of CELF2 dysregulation or one of many downstream targets contributing to immune imbalance?

Looking ahead, this study exemplifies a growing recognition that RNA processing is not noise but a signal in disease pathogenesis.^2^^,^^3^ The CELF2-CTSB axis may represent the first of many RNA-regulatory circuits uncovered in refractory autoimmune disorders. The ability to map and manipulate PAS usage heralds a new therapeutic horizon, where modulating transcript isoforms complements immunosuppression and biologic therapies.

Wei and colleagues^1^ shed light on the intricate interplay between APA and AS in refractory JDM, identifying CELF2 PAS usage as a pivotal regulatory event that dictates CTSB splicing, TNF signaling, and treatment outcomes. Their work bridges fundamental RNA biology with clinical immunology and opens new avenues for biomarker discovery and RNA-targeted therapy. While validation in larger cohorts is needed, the study paves the way for integrating RNA processing into the therapeutic vocabulary of autoimmune disease.

## Author contributions

R.V. and L.L. wrote the commentary.

## Declaration of interests

The authors declare no competing interests.
